# Eosinophilic Esophagitis in the Setting of Prior Caustic Ingestion

**DOI:** 10.7759/cureus.35536

**Published:** 2023-02-27

**Authors:** Marko Kozyk, Kateryna Strubchevska, Yana Kravchenko, Dariia Voroniak, Jennifer Cushman, Suprabhat Giri

**Affiliations:** 1 Internal Medicine, Beaumont Hospital, Royal Oak, USA; 2 Gastroenterology, National Specialized Children's Hospital «Ohmatdyt», Kyiv, UKR; 3 Internal Medicine, Michigan State University College of Osteopathic Medicine, Lancing, USA; 4 Gastroenterology and Hepatology, Nizam's Institute of Medical Sciences, Hyderabad, IND

**Keywords:** odynophagia, food allergy, chemical burn, dysphagia, eosinophilic esophagitis

## Abstract

A seven-year-old male presented with complaints of food refusal, dysphagia, and odynophagia for three weeks. He also had a history of caustic ingestion six months prior to the presentation. Diagnostic esophagogastroduodenoscopy (EGD) revealed post-burn esophageal stricture, and biopsy confirmed eosinophilic esophagitis (EoE). In this report, we discuss the diagnosis and management of these pathologies. We suspect that the damage sustained from caustic ingestion laid the groundwork for the development of EoE in this patient.

## Introduction

Chemical burns of the esophagus are considered life-threatening emergencies. The upper gastrointestinal tract is the region most commonly affected by chemical burns, and esophageal strictures can form weeks after the injury, causing dysphagia and odynophagia. However, eosinophilic esophagitis (EoE) can present in a similar manner. EoE is a chronic immune-mediated disease characterized by marked eosinophilic infiltration of the esophageal mucosa and can lead to fibrosclerotic changes due to inflammation and subsequent narrowing of the esophagus. We present a case of a child with esophageal stricture due to prior caustic ingestion, who was diagnosed with EoE on biopsy. Both conditions can cause nonspecific complaints in children, including dysphagia, pain with swallowing, and vomiting.

## Case presentation

A seven-year-old male presented to the hospital with complaints of food refusal, pain with swallowing, difficulty swallowing solids for three weeks, nausea, and vomiting for one week. The patient denied fever, chills, hematemesis, or weight loss. He did not have a past medical history of atopic comorbidities like asthma, atopic dermatitis, immediate food-type allergies, or allergic rhinitis.

Six months before the presentation, the child had burned his esophagus through ingestion of an aggressive alkaline substance (dishwasher liquid soap). An esophagogastroduodenoscopy (EGD) performed at that time at an outside hospital had revealed friability, erosions, and superficial ulceration. The patient did not obtain other medical records (laboratory and biopsy results) from the outside hospital. The patient had improved clinically and had been discharged home on an oral proton pump inhibitor for one week. The patient did not follow up at the outside hospital.

**Figure 1:**
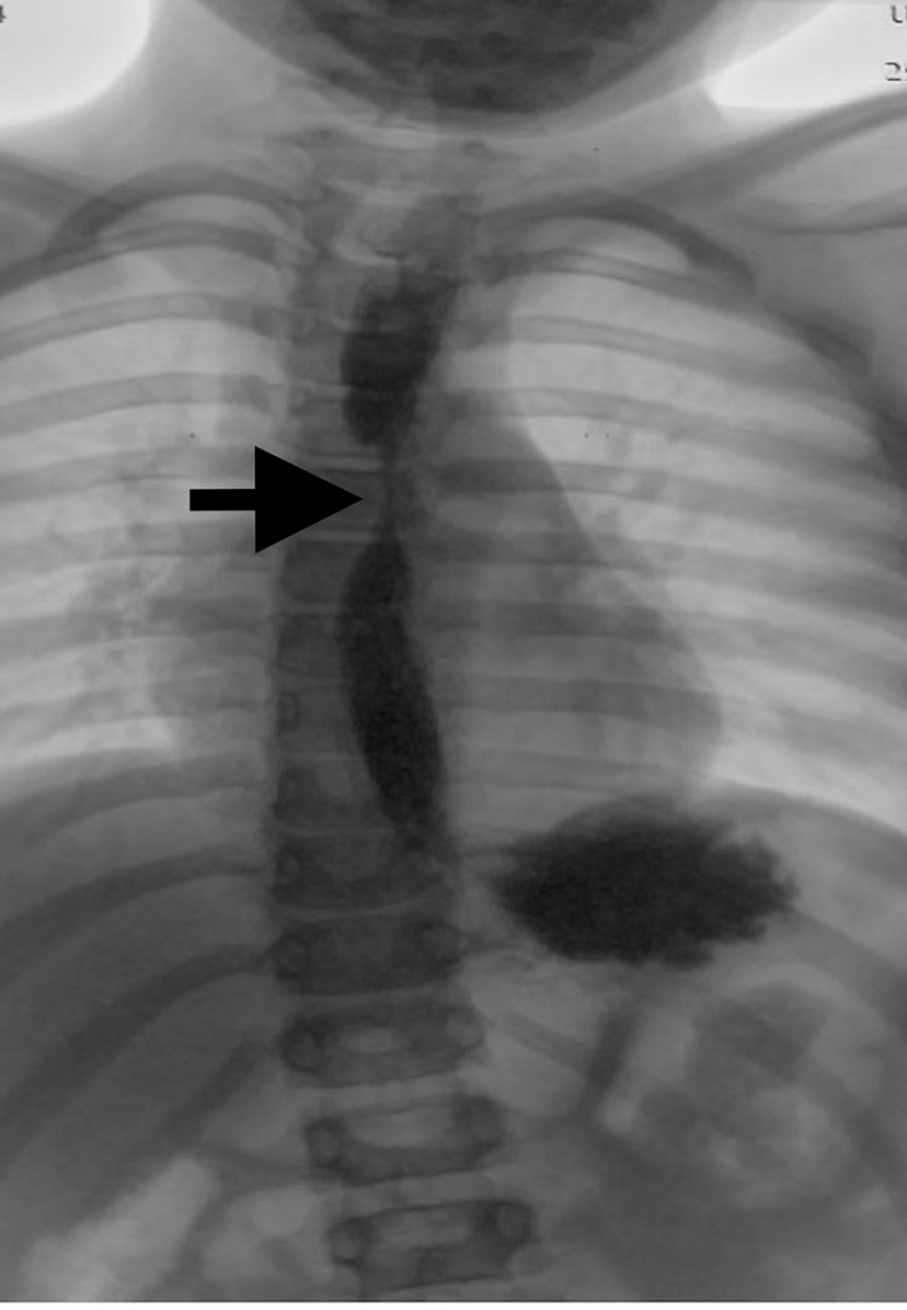
Contrast esophagram

Upon presentation to the National Specialized Children's Hospital «Ohmatdyt", the patient’s laboratory test results were significant for a differential eosinophil count of 5% (normal range: 1-4%). An esophageal narrowing was noted on the contrast esophagram (Figure 1).

**Figure 2:**
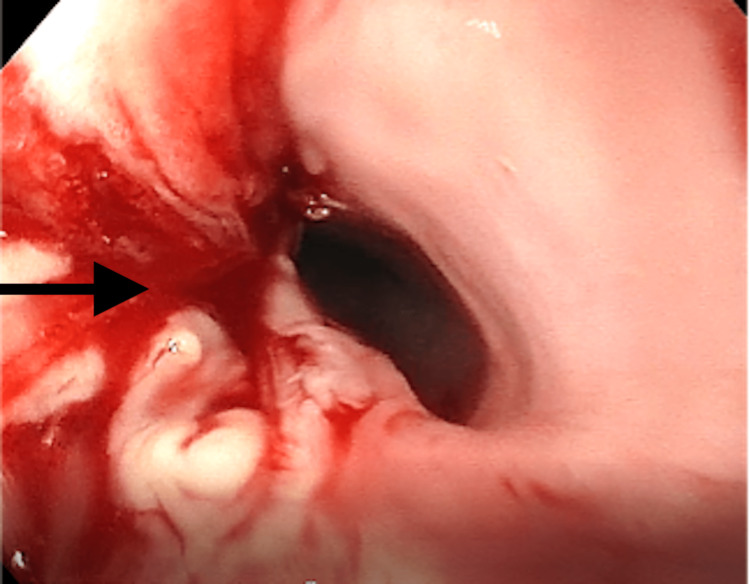
Endoscopic image revealing marked traumatization

EGD at the National Specialized Children's Hospital «Ohmatdyt" demonstrated a stenotic zone with inflammation and sticky fibrin patches. Endoscopic balloon dilation was performed to restore the patency of the esophagus. Marked traumatization of the mucous membrane upon contact with the endoscope was noted (Figure 2).

**Figure 3:**
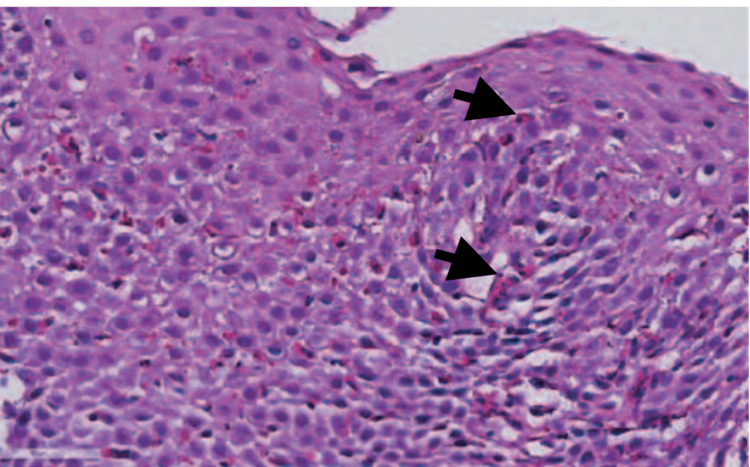
Diffuse eosinophilic infiltration of the stratified squamous epithelium with the presence of more than 15 eosinophils per high-power field

Random biopsies from the distal, mid, and proximal esophagus demonstrated diffuse eosinophilic infiltration of the stratified squamous epithelium with more than 15 eosinophils per high-power field, eosinophilic microabscesses, and basal zone hypertrophy (Figure 3).

The child was treated with esomeprazole 20 mg daily and a six-food elimination diet for EoE, eliminating dairy, wheat, soy, eggs, nuts, and seafood/shellfish from the diet. The patient refused to undergo food-allergy testing. He was found to have clinical and endoscopic improvement on follow-up visits.

## Discussion

Chemical burns of the esophagus are caused by caustic substances, which are widely available and can damage any segment of the gastrointestinal tract [1]. Most often, the upper segments of the gastrointestinal tract are affected. Damage to the gastrointestinal tract by chemical substances is observed with accidental or suicidal ingestion of acids or alkalis; accidental ingestion is a common cause in children, whereas suicidal intent is prevalent in adults [1]. The degree of the chemical burn depends on several factors, such as the chemical properties of the substance, dose, concentration, duration of action, and individual patient characteristics. It is crucial to provide prompt medical aid after a chemical burn so that the severity of the injury can be mitigated. First-degree burns affect only the mucous membrane and cause localized redness and edema, which can be observed with endoscopy. Second-degree burns involve the mucosa and submucosa, while third-degree burns are characterized by a transmural process with deep ulcers and areas of necrosis [2].

The most important diagnostic method for esophageal chemical burns is EGD, which can assess the presence of a lesion, its extent, depth, and degree [3]. Endoscopic diagnostics are used at all stages of treatment to monitor the course of the disease, the healing process, and the presence of scar stricture. Esophageal strictures begin to form three to four weeks after injury. The main symptoms are dysphagia and odynophagia [3]. We suspect that our patient's EoE was a result of caustic ingestion.

EoE, once considered a rare entity, is now one of the most common conditions diagnosed during the evaluation of dysphagia and food impaction in children and adults [4]. There has been a significant increase in the prevalence of EoE, which is associated with an increase in its incidence as well as an improvement in diagnostic methods. Disease prevalence among the asymptomatic adult population is estimated to be 0.4% [5]. Among children undergoing EGD for abdominal pain, the frequency of EoE was reported to be 4% [5]. Endoscopic features of EoE include circular rings, linear furrows, white plaques, and exudates [5]. EoE is a chronic immune-mediated disease characterized by marked eosinophilic infiltration of the esophageal mucosa (≥15 eosinophils per high-power field of view). Fibrosclerotic changes due to inflammation lead to the narrowing of the esophagus and dysphagia [6].

Symptoms of EoE are often nonspecific and vary with age. Abdominal pain, feeding difficulties, refusal to eat, and decreased body weight and height are common complaints in younger children. Older children typically present with dysphagia, discomfort behind the sternum, nausea, heartburn, a feeling of a lump in the throat, and chest pain [7]. The gold standard for the diagnosis of EoE is EGD, a biopsy of the esophageal mucosa, and histological evaluation.

In a previous case report, a patient who developed esophageal stricture due to EoE had a history of alkali ingestion one year prior, similar to the child in our case [8]. The authors hypothesized that caustic ingestion could be a risk factor for the development of EoE due to the breakdown of the esophageal mucosal barrier by the caustic material, which enables food antigens to trigger an immune response resulting in EoE.

## Conclusions

In the pediatric population, one must keep a wide differential when evaluating cases involving food refusal and difficulty swallowing, as their complaints may be nonspecific. Additionally, it is important to consider that there may be more than one underlying condition contributing to a patient’s presentation, as was seen in our case. Therapeutic balloon dilation may improve post-burn esophageal strictures; however, when superimposed by EoE, treatment with food elimination is also essential for the remission of symptoms. Other treatment options for EoE include topical steroids and biologics.
